# Withaferin A inhibits adipogenesis in 3T3-F442A cell line, improves insulin sensitivity and promotes weight loss in high fat diet-induced obese mice

**DOI:** 10.1371/journal.pone.0218792

**Published:** 2019-06-21

**Authors:** Manizheh Khalilpourfarshbafi, Dharmani Devi Murugan, Munavvar Zubaid Abdul Sattar, Yamuna Sucedaram, Nor Azizan Abdullah

**Affiliations:** 1 Department of Pharmacology, Faculty of Medicine, University of Malaya, Kuala Lumpur, Malaysia; 2 Faculty of Pharmacy, MAHSA University, Jenjarom, Malaysia; Weill Cornell Medical College in Qatar, QATAR

## Abstract

The increased prevalence of obesity and associated insulin resistance calls for effective therapeutic treatment of metabolic diseases. The current PPARγ-targeting antidiabetic drugs have undesirable side effects. The present study investigated the anti-diabetic and anti-obesity effects of withaferin A (WFA) in diet-induced obese (DIO) C57BL/6J mice and also the anti-adipogenic effect of WFA in differentiating 3T3- F442A cells. DIO mice were treated with WFA (6 mg/kg) or rosiglitazone (10 mg/kg) for 8 weeks. At the end of the treatment period, metabolic profile, liver function and inflammatory parameters were obtained. Expression of selective genes controlling insulin signaling, inflammation, adipogenesis, energy expenditure and PPARγ phosphorylation-regulated genes in epididymal fats were analyzed. Furthermore, the anti-adipogenic effect of WFA was evaluated in 3T3- F442A cell line. WFA treatment prevented weight gain without affecting food or caloric intake in DIO mice. WFA-treated group also exhibited lower epididymal and mesenteric fat pad mass, an improvement in lipid profile and hepatic steatosis and a reduction in serum inflammatory cytokines. Insulin resistance was reduced as shown by an improvement in glucose and insulin tolerance and serum adiponectin. WFA treatment upregulated selective insulin signaling (*insr*, *irs1*, *slc2a4* and *pi3k*) and PPARγ phosphorylation-regulated (*car3*, *selenbp1*, *aplp2*, *txnip*, and *adipoq*) genes, downregulated inflammatory (*tnf-α* and *il-6*) genes and altered energy expenditure controlling (*tph2* and *adrb3*) genes. In 3T3- F442A cell line, withaferin A inhibited adipogenesis as indicated by a decrease in lipid accumulation in differentiating adipocytes and protein expression of PPARγ and C/EBPα. The effect of rosiglitazone on physiological and lipid profiles, insulin resistance, some genes expression and differentiating adipocytes were markedly different. Our data suggest that WFA is a promising therapeutic agent for both diabetes and obesity.

## Introduction

Obesity and its associated metabolic disorders such as type 2 diabetes, dyslipidemia and cardiovascular diseases has become a global epidemic [1]. The exact molecular mechanisms linking obesity to insulin resistance is not completely understood and blood levels of free fatty acids (FFAs) appears to play a vital role in the development of obesity-associated insulin resistance [2]. Clinical studies have shown increased plasma FFA levels in most obese subjects and have associated it to insulin resistance [1, 3]. The mechanisms suggested to be involved in FFA-induced insulin resistance include accumulation of lipids and lipid intermediates, activation of several protein kinase C isoforms, activation of the pro-inflammatory nuclear factor-κB (NF-κB) pathway [4] and reduction in tyrosine phosphorylation of the insulin receptor substrates (IRS) 1 and 2 [1].

The nuclear receptor peroxisome proliferator-activated receptor-γ (PPAR-γ) is the master regulator of lipid and glucose metabolism and is abundantly expressed in various tissues including adipose tissue [5, 6]. Activation of PPARγ by different ligands such as fatty acids leads to adipocyte differentiation and fatty acid storage. PPARγ is also the site action of the anti-diabetic thiazolidinedione drugs [7]. Thiazolidinedione drugs including rosiglitazone strongly activate PPARγ and are potent insulin-sensitizing agents. Additionally, these drugs decrease expression of insulin resistance-inducing adipokines including TNF-α, IL-1 and resistin, and increase production of the insulin-sensitizing hormone, adiponectin [8, 9]. Although thiazolidinediones are highly effective oral medications for type 2 diabetes mellitus, their unique benefits are shadowed by the risk for adverse effects such as weight gain, fluid retention, bone loss and heart problems [10]. Medicinal plants have recently attracted attentions as source material for potential drug developments and many people are seeking natural therapies for management of obesity and obesity-related health problems. Therefore, identification of an insulin sensitizing agent with minimal side effects yet effective in modulating energy metabolism is critically needed.

Withaferin A (WFA), a steroidal lactone derived from *Withania somnifera* is a potent inhibitor of NF-κB, a key signaling molecule in the elaboration of the inflammatory response, and has been demonstrated to be a potent, safe, anti-inflammatory molecule [11]. Our group has shown that WFA protects endothelial cells against palmitic acid-induced insulin resistance and dysfunction through its anti-inflammatory effect [12]. In addition, a recent report has provided evidence that WFA treatment of mice with diet-induced obesity resulted in a lower food intake and a substantial reduction of body weight. The authors suggested that WFA acts centrally as a leptin sensitizer to promote weight loss [13]. Although WFA reduces body weight in diet-induced obese mice, the peripheral effects of WFA on weight regulation and the mechanisms underlying its insulin sensitizing effect in obesity related insulin resistance are yet to be unveiled. Therefore, this study sought to elucidate the insulin sensitizing and anti-obesity effects of WFA in diet-induced obese C57BL/6J mice and 3T3- F442A adipocytes.

## Results

### Effects of WFA treatment on body weight gain

**Fig 1:**
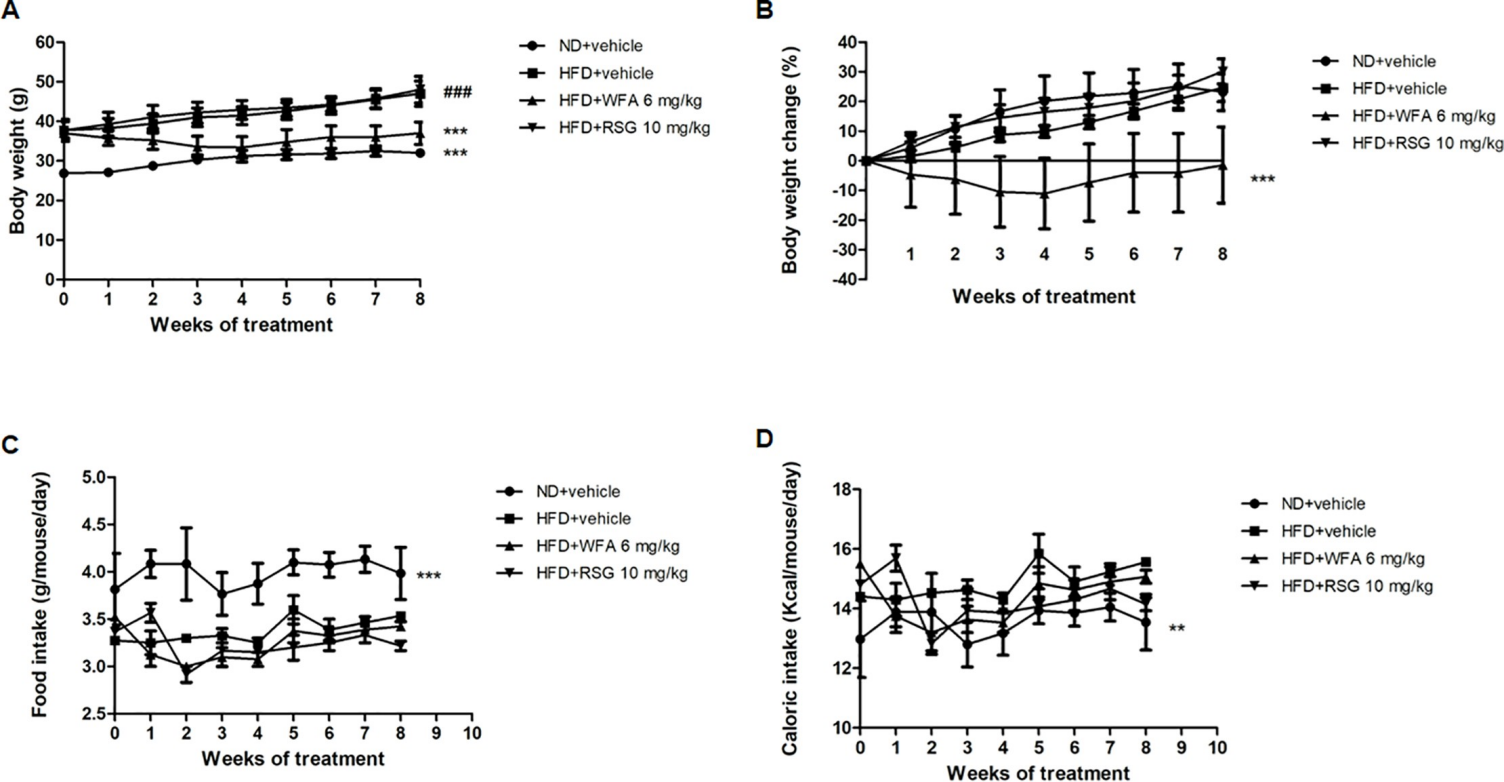
Effect of WFA treatment on diet-induced obese C57BL/6J mice. (A-D) Mice fed with ND received vehicle and mice fed with HFD received WFA (6 mg/kg/three times a week), RSG (10 mg/kg/day) or vehicle for 8 weeks. (A) body weight (B) percentage change in body weight. (C) daily food intake and (D) daily caloric intake. Values are represented as the mean ± SD, *n* = 8–10. **p < .01 and ***p < .001 vs HFD (vehicle) and ^###^*p* < .001 vs ND (vehicle).

To assess the effect of WFA on body weight, mice with DIO were treated with vehicle, WFA or RSG. The RSG and vehicle-treated groups continued to gain weight during treatment, equivalent to a total weight gain of 24.68 and 30.17% respectively, whereas WFA-treated group retained the same body weight throughout the treatment period (Fig 1A and 1B). However, there was no significant change in food (Fig 1C) or caloric intake (Fig 1D) in the WFA or RSG-treated groups compared to vehicle treated group.

### Effects of WFA treatment on adipose tissue mass and liver weight

**Fig 2:**
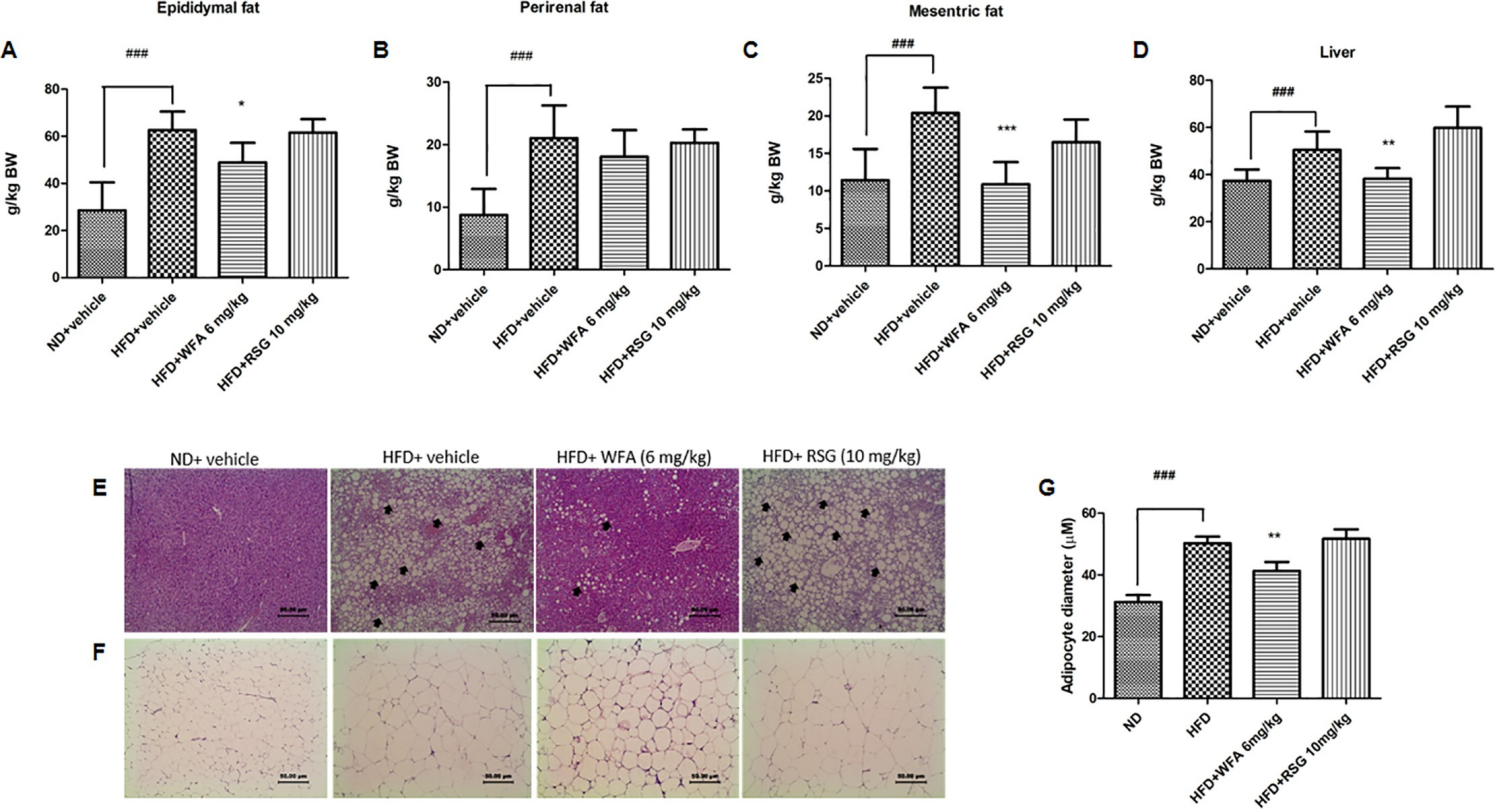
Effect of WFA treatment on liver and adipose tissue in diet-induced obese C57BL/6J mice. (A-G) ND group received vehicle and HFD group received WFA (6 mg/kg/three times a week), RSG (10 mg/kg/day) or vehicle for 8 weeks. The mass of (A) epididymal fat, (B) perirenal fat, (C) mesenteric fat, and (D) liver were normalized to body weight. Histopathological images of H&E stained sections of (E) liver and (F) adipose tissues. Values are represented as the mean ± SD, n = 8–10. *p < .05, **p < .01 and ***p < .001 vs HFD (vehicle) and ^###^p< .001 vs ND (vehicle). Black arrows indicate fat deposition in the mice livers. (Magnification: 400×, Scale bar: 50 μm) (E) and (F). Adipocyte size was measured using the ImageJ software with Adiposoft plugin (G).

Animals fed with ND exhibited lower perirenal, epididymal and mesenteric fat mass compared to HFD group. In the WFA-treated mice, there was a significant reduction in epididymal and mesenteric fat pad mass as compared to HFD group (Fig 2A–2C). However, no significant change was observed in perirenal fat pad mass after treatment with WFA. RSG treatment did not alter any of perirenal, epididymal or mesenteric fat pad mass. Mice fed with HFD had significantly increased liver weight compared with ND-fed mice (Fig 2D). WFA treatment but not RSG significantly reduced liver weight compared to their vehicle control group.

### Effects of WFA treatment on fat accumulation in liver and adipose tissue

Consumption of HFD led to hepatic steatosis as indicated by accumulation of lipids in the liver as compared with the ND group (Fig 2E). Histopathological assessment of the liver sections of WFA-treated mice showed a significant reduction in lipid globules when compared to HFD group. However, no significant improvement was observed in the liver of mice treated with RSG. As expected, HFD consumption caused adipocyte hypertrophy (Fig 2F). Treatment of obese mice with WFA significantly reduced the size of adipocytes when compared to HFD-fed mice (Fig 2G).

### Effect of WFA treatment on blood glucose, lipid profile and liver enzymes

Serum total cholesterol, triglyceride, glucose, ALT and AST levels were elevated in HFD-fed mice as compared with those on ND. Treatment with WFA and RSG significantly decreased triglyceride and glucose levels compared with vehicle control group while total cholesterol was only reduced in WFA-treated group (Table 1). Animals fed with HFD exhibited a marked elevation in ALT and AST levels. WFA treatment caused a significant reduction in ALT but not AST level. RSG treatment did not decrease the elevated liver enzymes (Table 1).

**Table 1:** Effect of WFA treatment on blood glucose, lipid profile and liver enzymes.

Parameters	ND+ vehicle	HFD+ vehicle	HFD+WFA	HFD+RSG
Fasting glucose (mmol/L)	6.00 ± 1.25	10.5 ± 1.2^###^	7.6 ± 1.2***	6.6 ± 1.06***
Triglyceride (mmol/L)	0.96 ± 0.26	1.35 ± 0.16^##^	0.69 ± 0.15***	0.95 ± 0.2**
Total cholesterol (mmol/L)	1.44 ± 0.31	4.26 ± 0.73^###^	2.69 ± 0.53***	4.37 ± 0.46
ALT (U/L)	30 ± 8.14	96 ± 31.47^###^	37 ± 16.09*	124 ± 34.30
AST (U/L)	93 ± 30.17	148 ± 41.79^##^	109 ± 15.35	123 ± 19.46

Fasting glucose, triglyceride, total cholesterol, ALT and AST of mice fed normal diet treated with vehicle or high-fat diet and treated with vehicle, 6 mg/kg WFA or 10 mg/kg RSG. Values are represented as the mean ± SD, n = 7–8.

*p < .05

**p < .01 and

***p < .001 vs HFD (vehicle).

^##^p < .001.

^###^p < .0001 vs ND (vehicle).

### Effect of WFA treatment on glucose homeostasis

**Fig 3:**
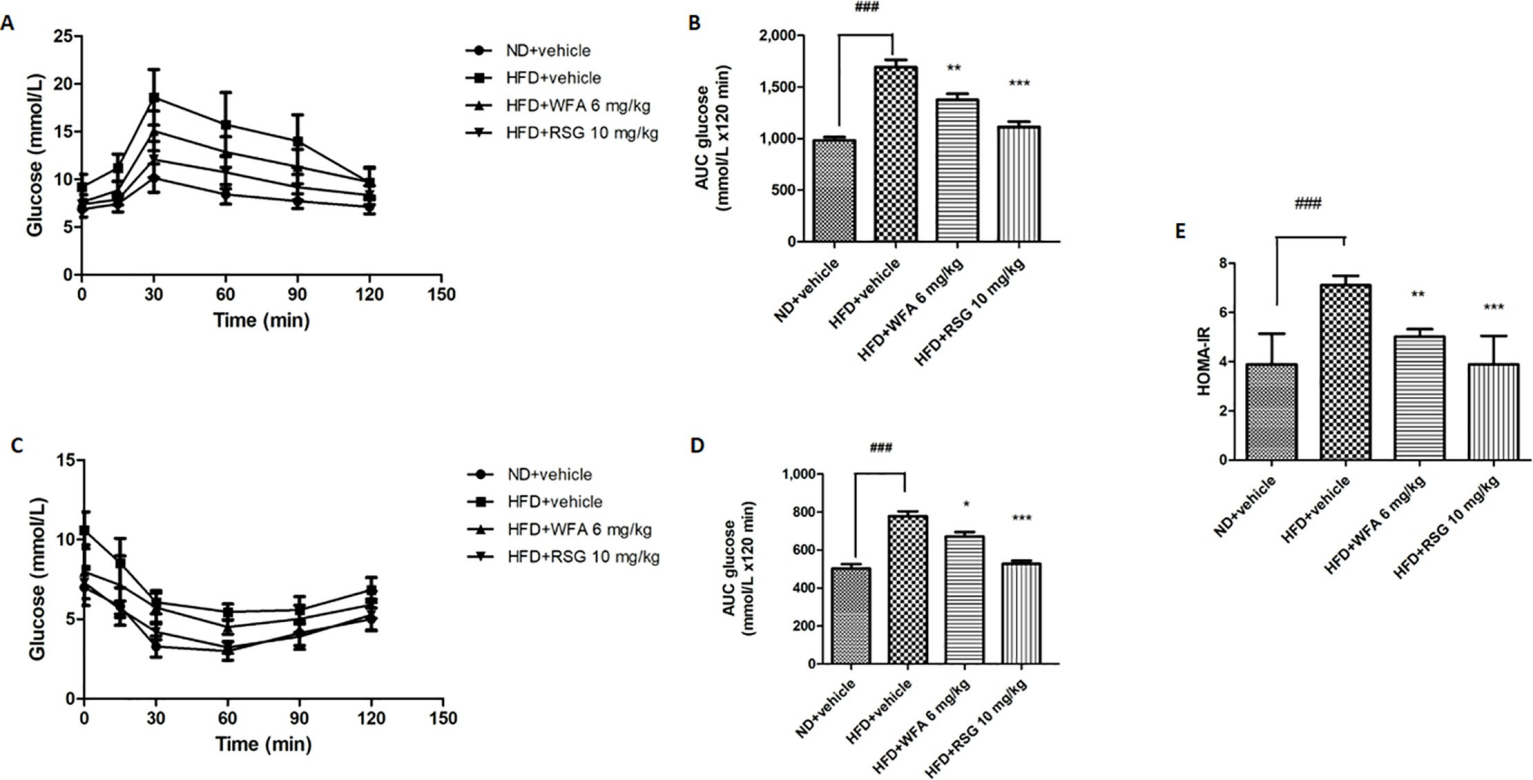
Effect of WFA treatment on glucose homeostasis in diet-induced obese C57BL/6J mice. (A-E) Mice fed with ND received vehicle and mice fed with HFD received WFA (6 mg/kg/three times a week), RSG (10 mg/kg/day) or vehicle for 8 weeks. (A) OGTT curve and its (B) area under the curve and (C) ITT curve and its (D) area under the curve and (E) insulin resistance index. Values are represented as the mean ± SD, n = 8–10. *p < .05, **p < .01 and ***p < .001 vs HFD (vehicle) and ^###^p < .001 vs ND (vehicle).

To assess the effect of WFA on glucose metabolism, oral glucose tolerance test was performed. HFD-fed mice exhibited impaired glucose tolerance as compared to ND-fed group. A significantly faster disposal of glucose from the circulation was observed in the WFA and RSG-treated mice, as compared to the vehicle-treated group (Fig 3A and 3B).

Insulin tolerance test revealed lower responsiveness to insulin in HFD group as compared to ND group. A significantly greater insulin tolerance was observed in WFA and RSG-treated mice compared to vehicle group (Fig 3C and 3D). In addition, the HOMA indexes were significantly lowered in WFA and RSG-treated mice as compared to HFD-fed mice indicating improvement in HFD-induced insulin resistance by both treatments (Fig 3E).

### Effect of WFA treatment on serum adipokines and insulin levels

**Fig 4:**
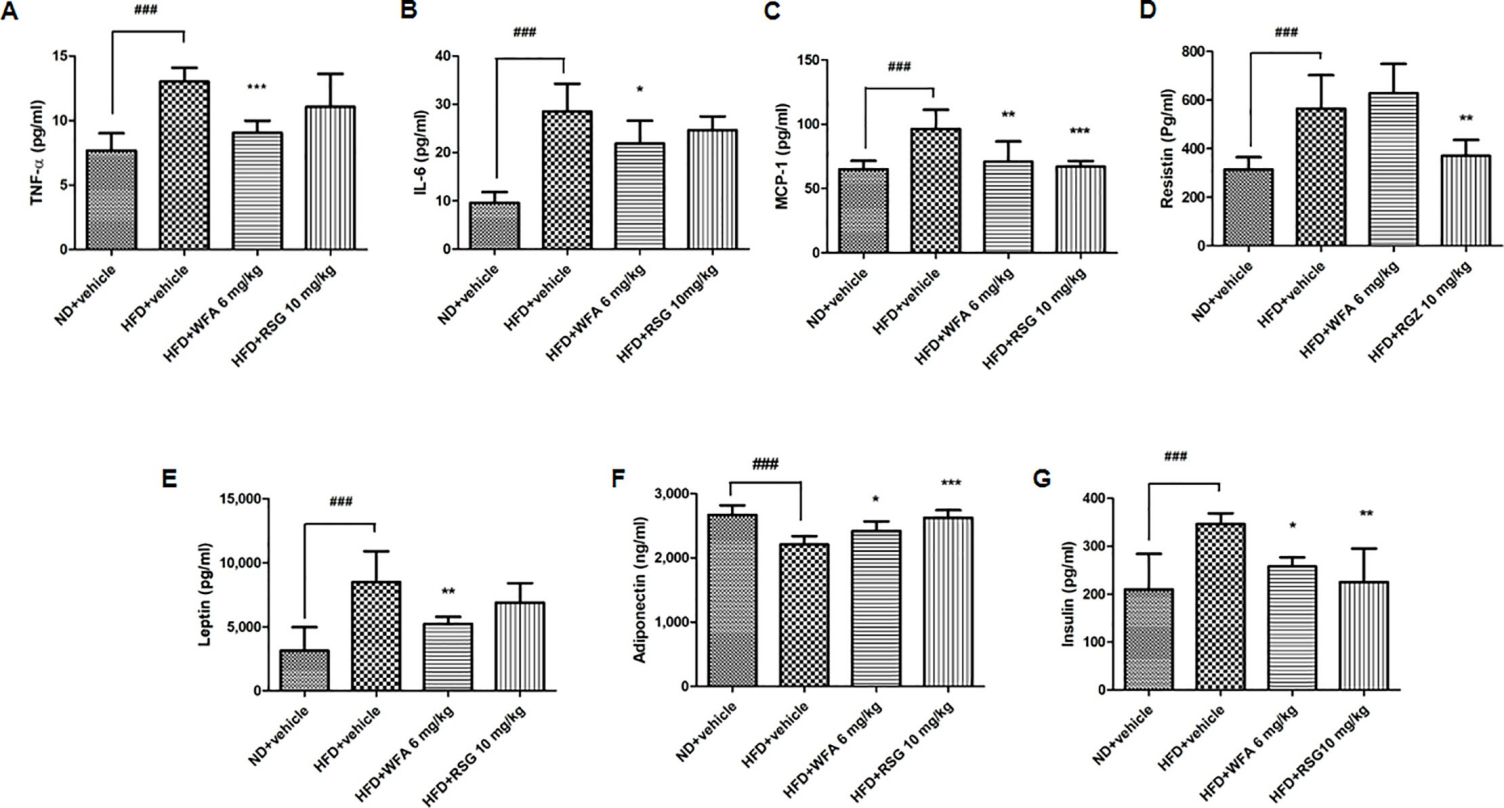
Effect of WFA treatment on serum adipokines and insulin in diet-induced obese C57BL/6J mice. (A-G) Mice fed with ND received vehicle and mice fed with HFD received WFA (6 mg/kg/three times a week), RSG (10 mg/kg/day) or vehicle for 8 weeks. Serum levels of (A) TNF-α, (B) IL-6, (C) MCP-1, (D) resistin, (E) leptin (F) adiponectin and (G) insulin. Values are represented as the mean ± SD, n = 8–10. *p < .05, **p < .01 and ***p < .001 vs HFD (vehicle) and ^###^p < .001 vs ND (vehicle).

Serum adipokines (TNF-α, IL-6, MCP-1, resistin, leptin) and insulin levels were elevated in HFD-fed mice compared with those on ND. WFA treatment significantly ameliorated the increased TNF-α, IL-6, MCP-1, leptin and insulin levels but did not affect resistin level (Fig 4). Animals received RSG showed a significant reduction in the levels of MCP-1, resistin and insulin but no significant change was observed in TNF-α, IL-6 and leptin levels (Fig 4). In contrast, adiponectin levels were markedly decreased in HFD-fed mice as compared to ND-fed animals and were increased following WFA and RSG treatment (Fig 4F).

### Effect of WFA treatment on genes controlling insulin signaling, inflammation, energy expenditure, adipogenesis and genes regulated by pparγ phosphorylation

Using a real-time PCR array, we evaluated the quantitative expressions of 58 genes controlling insulin signaling, inflammation, energy expenditure, adipogenesis and genes regulated by pparγ phosphorylation in the epididymal adipose tissue (Table 2). The HFD downregulated the expression of genes involved in insulin signaling (*insr*, *irs1*, *irs2 and pi3k*) with no significant changes in the expression of *slc2a4*. Treatment with WFA increased the mRNA expression of *insr*, *irs1*, *slc2a4* and *pi3k*, while RSG treatment increased the expression of *insr*, *irs1* and *slc2a4*.

**Table 2:** Fold change of selected genes in epididymal adipose tissue following WFA and RSG treatment in HFD animals.

Symbol	Group	Fold change	P value
**Insulin signaling**			
*insr*	ND	2.07	0.0000
	WFA	1.47	0.0009
	RSG	1.35	0.0006
*irs1*	ND	2.35	0.0000
	WFA	1.98	0.0003
	RSG	1.57	0.0705
*irs2*	ND	1.87	0.0034
	WFA	1.34	0.1021
	RSG	1.67	0.0024
*pi3k*	ND	1.64	0.3731
	WFA	2.73	0.0129
	RSG	2.23	0.1308
*slc2a4*	ND	1.15	0.2734
	WFA	1.35	0.0009
	RSG	1.38	0.0172
**Genes regulated by pparγ phosphorylation**			
*car3*	ND	3.80	0.0000
	WFA	1.57	0.0333
	RSG	1.42	0.1940
*nr1d1*	ND	2.05	0.0149
	WFA	1.01	0.9350
	RSG	0.86	0.3635
*cyp2f2*	ND	28.89	0.0041
	WFA	2.04	0.1881
	RSG	1.07	0.7253
*selenbp1*	ND	2.03	0.0018
	WFA	1.47	0.0595
	RSG	0.97	0.7333
*aplp2*	ND	1.76	0.0004
	WFA	1.35	0.010
	RSG	1.23	0.0295
*txnip*	ND	2.45	0.0000
	WFA	1.36	0.0390
	RSG	1.32	0.0483
*nr3c1*	ND	1.64	0.0052
	WFA	1.22	0.1879
	RSG	1.16	0.1719
*adipoq*	ND	1.44	0.0195
	WFA	1.53	0.0062
	RSG	2.34	0.0004
**Adipokines**			
*lep*	ND	0.26	0.0085
	WFA	0.53	0.0470
	RSG	0.89	0.5638
*cfd*	ND	14.70	0.0015
	WFA	2.24	0.2672
	RSG	1.57	0.8922
**Inflammation**			
*tnf-α*	ND	0.27	0.0379
	WFA	0.49	0.0251
	RSG	0.81	0.3144
*il-6*	ND	0.32	0.0008
	WFA	0.40	0.0018
	RSG	0.36	0.1087
*nfkb*	ND	1.03	0.6601
	WFA	1.09	0.4184
	RSG	1.11	0.1322
*ikkβ*	ND	1.31	0.0507
	WFA	1.16	0.0989
	RSG	1.14	0.0903
**Energy expenditure**			
*adrb3*	ND	6.11	0.0195
	WFA	1.95	0.0125
	RSG	2.34	0.0004
*faah*	ND	2.37	0.1803
	WFA	1.11	0.3541
	RSG	1.13	0.9863
*lipe*	ND	1.95	0.0001
	WFA	1.12	0.4722
	RSG	1.39	0.0608
*apoe*	ND	1.59	0.0057
	WFA	1.42	0.0327
	RSG	1.44	0.0180
*fasn*	ND	0.38	0.0172
	WFA	0.54	0.1601
	RSG	0.45	0.6979
*tph2*	ND	0.01	0.0024
	WFA	0.31	0.0444
	RSG	0.39	0.0611
**Adipogenesis**			
*pparγ*	ND	1.71	0.0022
	WFA	1.05	0.6571
	RSG	1.66	0.0089
*cebp-α*	ND	1.79	0.0243
	WFA	1.41	0.1400
	RSG	1.45	0.1324
*srebf1*	ND	1.14	0.3853
	WFA	1.18	0.2972
	RSG	1.55	0.0157

It has been reported that in obesity, phosphorylation of PPARγ at serine 273 stimulates diabetogenic gene expression in adipose tissues [14]. Therefore, we examined the mRNA expression of several genes regulated by PPARγ. The expression of *car3*, *nr1d1*, *cyp2f2*, *selenbp1*, *aplp2*, *nr3c1*, *txnip*, and *adipoq* was significantly decreased by HFD feeding. WFA-treated mice showed an upregulation of *car3*, *selenbp1*, *aplp2*, *txnip*, and *adipoq* while RSG treatment significantly increased *aplp2*, *txnip*, and *adipoq*.

The expression of several genes controlling adipokines were altered by HFD. The expression of *lep* was significantly increased while *adipoq* and *cfd* were decreased by HFD feeding. Both WFA and RSG treatment significantly upregulated *adipoq*. An increasing pattern in *cfd* expression was also observed in both treated groups, although not significant. In addition, WFA significantly lowered the HFD-induced overexpression of *lep*.

Furthermore, we investigated the effect of WFA and RSG on adipose tissue inflammation. The expression of genes encoding cytokines such as TNF-α and IL-6 were increased by HFD with no change in NFκB and IKKβ expression. WFA decreased the expression of these inflammatory cytokines. Similar expression pattern was observed in RSG-treated mice. However, these changes were not significant.

The mRNA expression of multiple genes controlling energy expenditure such as *adrb3*, *faah*, *lipe*, *apoe* decreased significantly by HFD feeding whereas, *fasn* and *tph2* were upregulated. Treatment with WFA downregulated *tph2* whilst both WFA and RSG treatment upregulated the expression of *adrb3*.

The mRNA expressions of adipogenic genes such as *pparγ* and *cebp-α* were increased by HFD feeding with no change in *srebf1*. WFA treatment had no effect on the expression of these genes while RSG treatment significantly increased *pparγ* and *srebf1* expression.

### Effect of WFA treatment on viability of preadipocytes and differentiating adipocytes

**Fig 5:**
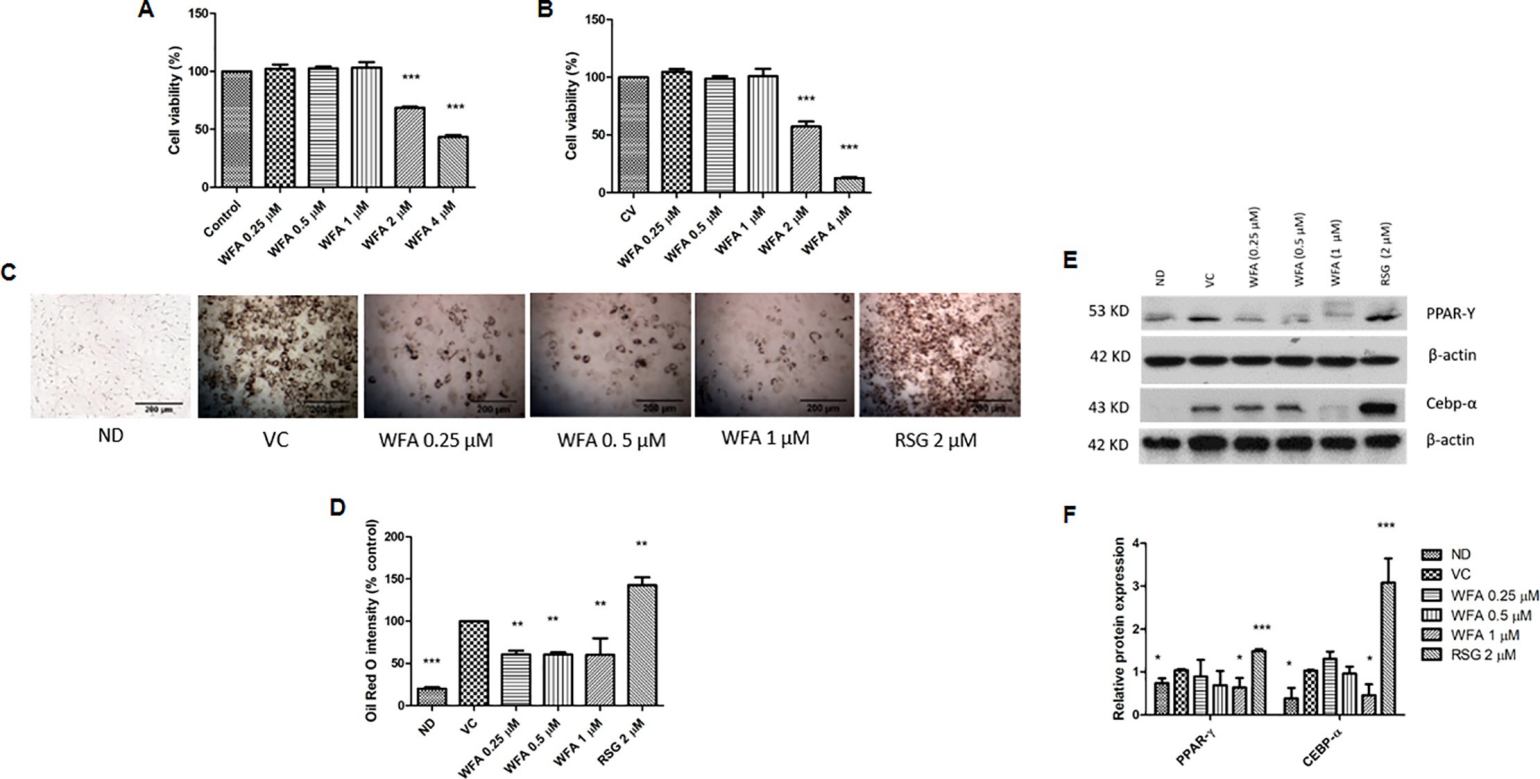
Inhibitory effect of WFA on adipogenesis. The effect of WFA on viability of (A) preadipocytes and (B) differentiating adipocytes. (C) Representative images of Oil Red O stained differentiating 3T3‐F442A preadipocytes treated with WFA (0.25–1 μM) and RSG (2 μM). Intracellular fat droplets were stained with Oil Red O and examined using light microscopy (magnification: 50x, scale bar: 200 μm). (D) The percentage of differentiation was calculated using the intensity of Oil Red O relative to that of control cells, assigned as 100% differentiation. (E) Protein expressions of PPARγ, and C/EBPα in differentiating 3T3-F442A cells on day 8 of differentiation. (F) The relative protein expression of PPARγ and C/EBPα. The values are represented as mean ± SD of three independent experiments. *p < .05, **p < .01 and ***p<0.001 vs vehicle control. ND = non differentiated cells, VC = vehicle control, WFA = withaferin A, RSG = rosiglitazone.

In order to investigate the effect of WFA alone on preadipocytes and differentiating adipocytes, the cells were treated with different concentrations of WA (0.25, 0.5, 1, 2 and 4 μM) for 8 days and assessed for the number of viable cells. WFA exerted cytotoxicity on adipocytes with an IC50 value of 2.88 ± 0.11 and 2.64 ± 0.31 μM in preadipocytes and mature adipocytes, respectively (Fig 5A and 5B). Therefore, the concentration ranges of 0.25–1 μM were selected for subsequent experiments.

### Effect of WFA treatment on adipogenesis

Adipogenesis is associated with an increase in intracellular lipid accumulation. The extent of adipocyte differentiation was visualized by staining the cells with lipid-specific stains and dye accumulation was quantified by absorbance readout. Eight-days of treatment of differentiating adipocytes with WFA (0.25, 0.5 and 1 μM) suppressed adipocyte differentiation and caused a significant reduction in lipid accumulation (50% inhibition) as compared to vehicle treated cells Nevertheless, treatment of the cells with RSG (2 μM) significantly increased adipocyte differentiation as indicated by increased numbers of cells with lipid droplets and increased dye intensity as shown in Fig 5C and 5D.

Adipogenesis is tightly controlled by the activity of transcription factors. The key transcription factors involved in adipogenesis include C/EBPα and PPARγ which positively control each other and cooperate to orchestrate expression of the full adipogenic program [15]. Therefore, the effect of WFA treatment on protein expression of these important transcription factors in differentiating adipocytes was evaluated. The protein expressions of C/EBPα and PPARγ on day 8 of differentiation were significantly reduced with 1 μM WFA treatment indicating anti-adipogenic effect of WFA (Fig 5E and 5F). In contrast, treatment of differentiating cells with RSG promoted adipogenesis by increasing the expression of these proteins.

## Discussion

Obesity is strongly associated with hyperglycemia and insulin resistance, and thiazolidinedione drugs have been widely used in the management of insulin resistance. However, these drugs can have long-term adverse effects including weight gain and other disorders, through their agonistic actions on PPARγ [10]. A previous study has demonstrated that WFA improves insulin sensitivity and promotes weight loss by acting as a leptin sensitizer [13], while the present study investigated further the insulin sensitizing and anti-obesity effects of WFA.

Our results clearly demonstrates that WFA induces beneficial changes in glucose metabolism and lipid profiles, attenuates inflammation and promotes weight loss in DIO mice, all of which contribute to increased insulin sensitivity. Additionally, WFA treatment ameliorates hepatic steatosis in these mouse models. In contrast, although rosiglitazone improves insulin sensitivity, the antidiabetic drug is devoid of liver protective and weight beneficial effects.

Obesity is associated with inflammation and inflammatory cytokines including TNF-α, IL-6 and resistin promote phosphorylation of insulin receptor substrates 1 (IRS-1) at serine sites that negatively regulates normal insulin signaling [16, 17]. Our previous study has reported that WFA restores impaired insulin resistance in cultured endothelial cells by suppression of inflammation and thus prevents phosphorylation of IRS-1 [18]. In the present results, adipose tissue mRNA and serum levels of IL-6, TNF-α and MCP-1 were elevated in DIO mice which were significantly reduced by WFA treatment. Furthermore, studies have demonstrated that obesity-induced insulin resistance and type 2 diabetes are associated with altered gene expression of insulin signaling genes [19]. Indeed, in the present study, the HFD downregulated the gene expression of insulin signaling genes (*insr*, *irs1*, *irs2 and pi3k*) with no significant changes in the expression of *slc2a4*. WFA treatment of mice with DIO increased the mRNA expression of *insr*, *irs1*, *slc2a4* and *pi3k*, while rosiglitazone treatment increased the expression of *insr*, *irs1* and *slc2a4*. These results indicate that WFA increased *insr* and *irs-1* levels, thereby enhancing insulin effects on IRS-1-dependent PI3K and downstream signaling. The *scl2a4* encodes a protein that functions as an insulin-regulated facilitative glucose transporter [20]. Thus, we postulate that the anti-inflammatory effect of WFA may contribute to its enhanced insulin action and translocation of GLUT4 to cell membrane thereby increasing glucose uptake in DIO mice.

Peroxisome proliferator activated receptor gamma (PPARγ) is a nuclear receptor that controls glucose and lipid metabolism [21]. High fat diet causes the activation of protein kinase CDK5 and ERK which are downstream of inflammatory pathways and are regulated by oxidative stress, cytokines and free fatty acids [22]. CDK5/ ERK axis causes the phosphorylation of PPARγ at serine 273. This phosphorylation neither stimulates nor represses PPARγ activation but rather dysregulates a specific set of genes with roles in obesity and diabetes [23–25]. It is well-known that the anti-diabetic effect of thiazolidinediones such as rosiglitazone is partly due to inhibition of PPARγ phosphorylation at serine 273 [22]. Our previous study demonstrated that WFA suppresses inflammation by inhibiting nuclear factor (NF)-κB activation [12]. Thus, we speculate that through inhibition of inflammation, WFA may prevent phosphorylation of PPARγ at serine 273 albeit we cannot rule out that WFA may act directly by docking to PPARγ at serine 273. Adiponectin is an adipocyte-specific protein which is dominantly regulated by PPARγ at the transcriptional level [26]. This adipokine improves insulin sensitivity by regulating GLUT4 translocation, fatty acid transport and breakdown [27–29]. It also stimulates ß-oxidation and glucose uptake via activation of adenosine monophosphate activated protein kinase (AMPK) [28]. Although adiponectin is produced by adipocytes, the serum levels of adiponectin decreases with obesity [30]. In the present study, the HFD reduced the white adipose tissue mRNA and serum levels of adiponectin. Indeed, treatment with both WFA and rosiglitazone increased significantly both mRNA and serum levels of adiponectin. In addition to adiponectin, phosphorylation of PPARγ at serine 273 causes dysregulation of several other genes in adipose tissue. Therefore, we studied the expression of several other genes that are regulated by PPARγ phosphorylation. Among them, the mRNA expression of *car3*, *nr1d1*, *cyp2f2*, *selenbp1*, *aplp2*, *nr3c1*, *txnip*, and *adipoq* were decreased significantly by HFD. WFA-treated mice showed upregulation of *car3*, *selenbp1*, *aplp2*, *txnip*, and *adipoq* whilst rosiglitazone upregulated *aplp2*, *txnip*, and *adipoq*.

Importantly and in contrast to rosiglitazone, our results indicates that WFA treatment of mice with DIO not only improved insulin sensitivity but also reduced epididymal adipocyte size, epididymal and mesenteric fat pad mass, decreased lipid accumulation in the liver and promoted weight loss. In addition, obesity is associated with hypertriglyceridemia and hypercholesterolemia as there is a significant correlation between impaired lipid metabolism and obesity [31]. The results from the present study showed lower serum concentrations of triglyceride and total cholesterol levels in WFA-treated obese mice, further supporting its effect on weight regulation. The question now arises whether the improvement in glucose homeostasis by WFA could be merely a secondary response to weight loss in our mouse models. However, our previous study which demonstrated that WFA improves insulin resistance in cultured endothelial cells support the notion that the effect of WFA could also be independent of weight loss [18].

There are several potential mechanisms by which WFA may affect weight regulation. A recent study showed that WFA causes weight loss in diet induced obese mice due to its leptin sensitizing effect through reduction of endoplasmic reticulum stress in hypothalamus [13]. The satiety hormone leptin is produced by adipocytes in direct proportion to their triglyceride content which leads to diminished food intake by binding to and activating its receptor in the brain [32]. Obesity promotes various pathways of cellular leptin resistance therefore restoring leptin sensitivity causes weight loss by affecting appetite. However, in our study WFA treatment did not show any decrease in food intake or calorie consumption and the significantly lower serum leptin levels in WFA treated group seems to be secondary to their lower fat mass as the serum leptin levels were directly proportional to body fat mass. The reason for the discrepancy is not known but the duration and frequency of WFA treatment differ in these studies. On the contrary, WFA may also act by other mechanisms to promote weight loss that are independent of its leptin sensitizing effect. Indeed Lee *at al*. (2016) reported that WFA treatment of *ob*/*ob* and *db*/*db* mice which lack leptin and leptin receptors respectively also led to a small but significant weight loss [13].

Recent studies have reported that a prolonged period of obesity may lead the body to recruit new preadipocytes and stimulate their differentiation into mature adipocytes, increasing the number of total adipocytes [33–35]. Adipogenesis is tightly controlled by the activity of a complex network of transcription factors which include c/ebpα and pparγ [35]. Rosiglitazone is a PPARγ agonist and promotes adipogenesis in *in vitro* and increases weight gain in rodent and humans [36–38]. Therefore, an agent that possesses insulin sensitizing effect without stimulating adipogenesis can be advantageous in the management of insulin resistance.

Next, we analyzed the expression of differentiation genes that are specifically important to adipose tissue in our mouse models of obesity. Our results however showed a significant decrease in the mRNA expression of *c/ebpα* and *pparγ* in HFD-fed mice. This result is in consistent with a previous report which showed decreased expression of these genes in obese mice as compared to lean mice [39]. The decreased in expression may be an attempt by the adipocyte to restrict fat accumulation and/or may merely be indicative of the cells being large and insulin resistant [40]. Alternatively, this could be due to depleted expression of adipogenic genes particularly *c/ebpα* and *pparγ* in mature adipocytes [41] suggesting that some degree of adipogenesis may have already taken place. Therefore, we examined the effect of WFA on adipogensis in 3T3-F442A cells during differentiation. Several studies have demonstrated that downregulation of C/EBPα and PPARγ expression during differentiation prevents mature adipocyte formation and lipid accumulation in 3T3-L1 preadipocytes [42–46]. We have selected concentrations of 1μM and below, which in accordance to our MTS assay do not affect the viability of the cells, to study the anti-adipogenic effect of WFA in 3T3-F442A cell line. We acknowledge that WFA is toxic to many cancer cell lines at lower concentrations, however the IC50 values are significantly less cytotoxic to normal cell lines [47, 48] suggesting that WFA is selectively toxic to cancer cells. In support, our previous study showed that WFA at concentrations of less than 8μM is not only non-toxic to human umbilical vein endothelial cells (HUVECs) but also increases the viability of these cells [12].

Our finding showed WFA treatment at selected concentrations was not toxic to preadipocytes or differentiating adipocytes but was potent in inhibiting adipogenesis as indicated by reduced protein expression of C/EBPα and PPARγ. On the other hand, as expected, treatment of differentiating cells with rosiglitazone upregulated the expression of PPARγ and C/EBPα which is indicative of enhanced adipogenesis.

Regulation of energy expenditure is an important aspect of weight reduction. β-adrenergic receptors play an important role in the regulation of lipolysis in human adipose tissue and provides free fatty acids for thermogenesis [49]. Studies have reported that HFD decreases energy expenditure and the expression of adrenoceptor beta 3 (*adrb3*) is significantly lower in obese patients [49]. Additionally, individuals with a low ADRB3 function in adipose tissue gain weight, whereas a high receptor function seems to protect from weight gain [50]. Tryptophan hydroxylase 2 (*Tph2*) is another energy expenditure controlling gene that increases significantly by HFD in adipose tissue [51]. Deficiency of Tph2 in mice causes body fat reduction by increasing energy expenditure [52]. Although the expression of *tph2* increases significantly by HFD feeding, the specific functions of this gene in the adipose tissue are still unclear. Notably, our gene expression analysis of adipose tissue in mice with DIO demonstrated that expression of genes associated with energy expenditure was altered in this study. In our study, the mRNA expression of *adrb3* was significantly decreased whilst the expression of *tph2* was significantly increased by HFD. Both WFA and rosiglitazone treatment increased the expression of *adrb3* in the obese animals, however only WFA decreased significantly the expression of *tph2*. These results suggest that WFA may positively affect energy expenditure although this postulation can only be confirmed by studies measuring oxygen consumption, carbon dioxide production and food intake.

We recognize of the possibility that the weight loss induced by WFA (18mg/kg/week) in our study could arise from the toxic effect of the drug. However, this assumption is unlikely to be valid since no toxicity was observed for the treatment period as confirmed by histopathological studies in the liver and biochemical analysis. In support, other authors claim that the weight loss in WFA-treated (17.5 mg/kg/week) obese C57BL/6J mice is not due to the toxic effect of the treatment [13]. The reason put forward by the authors is that lean animals treated with WFA do not show body weight and fat or lean mass changes as confirmed by DEXA scans. In addition, blood chemistry analyses (ALT, AST and thyroid hormones) in these animals are unaltered as compared to lean animals treated with vehicle.

In conclusion, we found that WFA improves insulin sensitivity and promotes weight loss. The insulin sensitizing effects of WFA seems to be through its anti-inflammatory effect which then affects insulin signaling pathway, increasing adiponectin, and also prevention of PPARγ phosphorylation. The anti-obesity effect of WFA could be through increasing energy expenditure, anti-inflammatory and anti-adipogenic effects. These results may herald the development of this drug for treatment of both obesity and diabetes.

## Materials and methods

### Animal experiments

Four-weeks old male C57BL/6J mice were housed under standard environmental conditions at a room temperature of 24±1°C with 12 h light/dark cycle in the AAALAC International accredited animal facility at the Department of Pharmacology, Faculty of Medicine, University of Malaya. All experimental protocols were approved by the Institutional Animal Care and Use Committee of Faculty of Medicine, University of Malaya (2016-190908/PHAR/R/NAA).

The animals were fed with standard laboratory chow for 2 weeks to allow them to adjust to the new environment. After 2 weeks, they were fed with normal (ND) or high fat diet (HFD) (45 kcal% from fat) for 10 weeks. After 10 weeks, animals were randomized into the following groups of treatment and continued to be fed with their pre-designed diets for another 8 weeks (n = 8 for each group): ND control group, HFD control group, and HFD+ WFA (isolated from *Withania somnifera*, sc-200381, Santa Cruz Biotechnology, USA) (6 mg/kg three times a week, i.p.), and HFD+ rosiglitazone (RSG) (10 mg/kg/day, orally). ND and HFD control groups were administered the vehicle, dimethyl sulfoxide (DMSO) (12 μl, i.p.). Body weights and food intake were recorded every 2 days. Oral glucose tolerance test and intraperitoneal insulin tolerance test were conducted on week 17. At the end of the 18 weeks period, animals were fasted for 6 h and blood samples were collected via cardiac puncture for biochemical analysis. The animals were sacrificed by carbon dioxide inhalation and visceral adipose tissue and liver were collected for gene expression and histopathological studies.

### Oral glucose tolerance test (OGTT)

Tail blood glucose was measured in all groups at 6 h post-fasting using an Accu‐check glucometer (Roche Diagnostics, Germany). D-glucose (2 g/kg) was administered orally and blood glucose was measured at 15, 30, 60, 90, and 120 min after glucose administration.

### Intraperitoneal insulin tolerance test (IPTT)

Tail blood glucose level was measured at 4 h post-fasting using an Accu‐check glucometer (Roche Diagnostics, Germany). Insulin (0.75 U/kg, i.p) was injected intraperitoneally and blood glucose levels were measured at 15, 30, 60, 90 and 120 min post-injection. In addition, the insulin resistance index (HOMA-IR) was calculated from fasting serum insulin and glucose levels, using the computer HOMA2 model (available from www.dtu.ox.ac.uk/homacalculator/).

### Serum biochemistry

Collected serum samples were sent to Hematology & Biochemistry Clinical Laboratory, Veterinary Laboratory Service Unit, Faculty of Veterinary Medicine, University Putra Malaysia, for measurement of fasting blood glucose, total cholesterol, triglycerides, aspartate aminotransferase (AST) and alanine aminotransferase (ALT) using Dimension Xpand Plus clinical chemistry system (SIEMENS Healthcare Diagnostics Inc.).

Levels of different adipokines (TNF-α, monocyte chemotactic protein 1 (MCP-1), leptin, insulin, interleukin-6 (IL-6) and resistin) in mouse sera were measured using the MILLIPLEX MAP Mouse Adipokine Magnetic Bead Panel (Millipore, Billerica, MA). Serum adiponectin level was measured by commercially available mouse adiponectin ELISA kit, (Millipore, Billerica, MA) according to manufacturer’s protocol.

### Histopathological analysis

Adipose tissues and liver were fixed in 10% buffered formalin, dehydrated and processed using an automated tissue processor (Leica, Germany). Four micrometer-thick sections were stained with haematoxylin and eosin (H&E) to examine the morphology using an automated slide strainer (Leica, Germany). Images were obtained under bright field LEICA DM 2000 microscope with LEICA ICC50 HD camera (Leica Microsystems, Germany). The diameter of adipocytes was measured using the ImageJ software with Adiposoft plugin [53].

### Quantitative real-time PCR

For gene expression studies, total RNA was extracted from epididymal adipose tissue using the RNeasy lipid tissue mini kit (Qiagen, Germany). The concentration and the purity of the extracted RNA was assessed using NanoDrop 2000/2000c spectrophotometer (Thermo Scientific, USA). The RNA was then reverse transcribed using RT2 strand kit (Qiagen,Germany). The mRNA expression of the genes involved in insulin signaling, inflammation, adipogenesis, energy expenditure, and genes regulated by the phosphorylation of PPARγ (ser 273) were determined on a custom RT2 profiler PCR array using SYBR Green qPCR Mastermix (Qiagen, Germany). Real time PCR was performed in a 96-well format thermal cycler (Biosystem StepOne Plus, Applied Biosystems, USA). The fold change in target gene expressions was determined following the 2¯ΔΔCt method.

### Cell culture

Mouse 3T3-F442A preadipocyte cells were obtained from European Collection of Cell Cultures (ECACC) and cultured in high glucose Dulbecco’s Modified Eagle Medium (DMEM) supplemented with 10% (vol/vol) calf serum, 100U/mL penicillin and 100U/mL streptomycin. The cells were maintained at subconfluency to prevent differentiation. The cells were seeded at 3.3 x 10^3^ cells/cm^2^ and maintained in DMEM/high glucose containing 10% calf serum until they were confluent. Differentiation was induced by adding differentiation media (DMEM/ high glucose, 10% fetal bovine serum (FBS), 5 μg/ml insulin, 0.4 μg/ml dexamethasone and 5 mM MIX). On day 2, DMEM/high glucose containing 10% FBS, 5 μg/ml insulin was added to the cells for further 2 days. The medium was then changed to DMEM/high glucose supplemented only with 10% FBS for an additional 4–6 days. Successful differentiation of the cells was confirmed by accumulation of lipid droplets using oil red O staining.

### Cytotoxicity test

Cell viability and cytotoxicity of WFA was determined using CellTiter 96 Aqueous One Solution Cell Proliferation Assay kit from Promega (Madison, WI) for the 3-(4,5-dimethylthiazol-2-yl)-5-(3-carboxymethoxyphenyl)- 2-(4-sulphophenyl)-2H-tetrazolium (MTS) assay according to the manufacturer instructions.

Preadipocytes were seeded in 96-well plates and incubated with either DMSO (1:1000) or increasing concentrations of WFA for up to 8 days. To assess the effects of WFA on the viability of differentiating adipocytes, 2 days postconfluent cells were induced to differentiation and co-treated with increasing concentrations of WFA for 8 days. At the end of treatment, 20 μl of MTS solution was added to each well and incubated for another 2 h. Finally, the absorbance was measured at a wavelength of 490 nm. The effect of WFA on cell viability was assessed as the percentage of cell viability compared to control cells, which were assigned with 100% viability.

### Oil Red O Staining and Measurement of Lipid Accumulation

Differentiating cells were incubated with either DMSO (1:1000) or increasing concentrations of WFA (0.25, 0.5 and 1 μM) for up to 8 days. The cells were stained, and lipid accumulation was measured. Briefly, the cells were fixed in 10% formalin for 1 h and then stained with Oil Red O solution (three parts 0.5% Oil Red O dye in isopropanol to two parts water) for 1 h. The dye solution was then removed and the cells were washed three times with 60% isopropanol to remove unbound dye. The images were obtained under light microscopes at 50× magnification (Axiovert 40 CFL, Carl Zeiss, Germany). Intracellular Oil Red O was extracted with isopropanol, and quantified by measuring the absorbance at the wavelength of 492 nm. The level of differentiation was determined by comparison of absorbance to those of control cells, which were assigned as 100% differentiation.

### Western blot analysis

Differentiating cells were treated with either DMSO (1:1000) or different concentrations of WFA (0.25, 0.5 and 1 μM) for 8 days. Total protein was extracted from the cells on day 8 of differentiation using RIPA lysis buffer containing protease inhibitor (cOmplete, Roche Applied Science) and phosphatase inhibitor (PhosSTOP, Roche Applied Science). The protein concentration was quantified using DC Protein Assay Kit (Biorad, USA). Fifteen microgram of extracted protein was mixed with loading dye, denatured and separated by SDS‐PAGE (2 h, 110 V), and then transferred (90 min, 110 V) to a polyvinylidene difluoride membrane (Millipore, USA). The membranes were blocked in 3% bovine serum albumin (Santa Cruz, USA) at room temperature for 1 h and incubated with the primary antibodies against PPARG (1:500, mouse monoclonal, Santacruz, #sc-7273) and CEBP/α (1:1,000, rabbit polyclonal, Santacruz, #sc-61). Membranes were then washed and incubated with respective horseradish peroxidase-conjugated secondary antibodies (DAKO, Denmark) for 2 h at room temperature. The protein bands were visualized with enhanced chemiluminescence substrate (Menlo Park, CA, USA) and exposed to X‐ray film (Fujifilm, Japan). Signals were quantified using Image Studio Lite version 4.0.21 (LI‐COR Biosciences) software and relative protein expression was determined by comparing the intensity of the band to those of the vehicle control.

### Statistical analysis

Data are expressed as mean ± standard deviation (SD) from n number of experiments and analyzed by one-way analysis of variance followed by Bonferroni's multiple comparison tests using Graph Pad Prism (Version 5.0, Graph Pad Software Inc., San Diego, CA). P value < .05 was considered to be statistically significant.

## Supporting information

S1 Fig Inhibitory effect of WFA on adipogenesis in differentiating 3T3‐F244A cells treated WFA (0.25–1 μM) and RSG (2 μM). The results are from three independent experiments. (a-c) Intensities of the PPARγ, and (d-f) C/EBPα protein bands were normalized to those of β‐actin, and relative protein expressions of the treated samples were obtained by comparing the normalized protein bands intensity to that of the vehicle control.
(PDF)
