# Supplementary material for: Withaferin A inhibits adipogenesis in 3T3-F442A cell line, improves insulin sensitivity and promotes weight loss in high fat diet-induced obese mice
Source: PLoS One. 2019 Jun 21;14(6):e0218792. doi: 10.1371/journal.pone.0218792 (PMC6588247; doi:10.1371/journal.pone.0218792)
Supplement: S1 Fig — The results are from three independent experiments. (a-c) Intensities of the PPARγ, and (d-f) C/EBPα protein bands were normalized to those of β‐actin, and relative protein expressions of the treated samples were obtained by comparing the normalized protein bands intensity to that of the vehicle control. (PDF) [file pone.0218792.s001.pdf]

S1 Appendix : original blots of westernblotting results of PPAR-γ and cebp-α

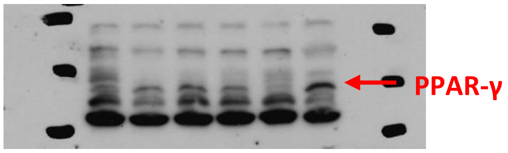

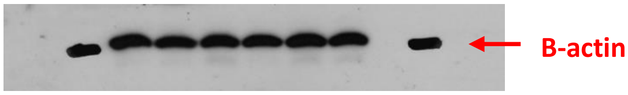

(PPAR-γ, n=1, 1:500, mouse monoclonal, Santacruz, #sc-7273)

(β-actin, n=1, 1:5,0000, mouse monoclonal, Santacruz, #sc-56459)

(l1=ND, l2=control, l3=WFA 0.25μM, l4= WFA 0.5μM, l5= WFA 1μM, l6=RSG 2 μM)

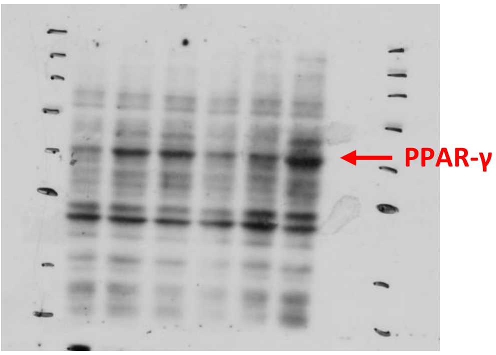

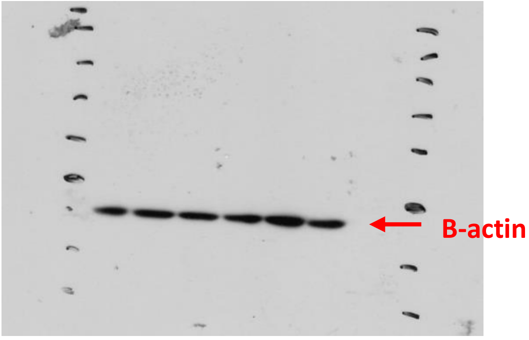

(PPAR-γ, n=2, 1:500, mouse monoclonal, Santacruz, #sc-7273)

(β-actin, n=2, 1:5,0000, mouse monoclonal, Santacruz, #sc-56459)

(l1=ND, l2=control, l3=WFA 0.25μM, l4= WFA 0.5μM, l5= WFA 1μM, l6=RSG 2 μM)

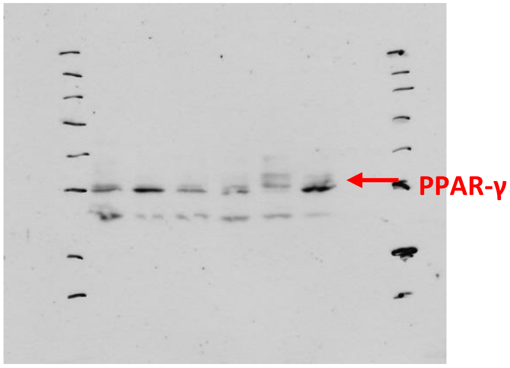

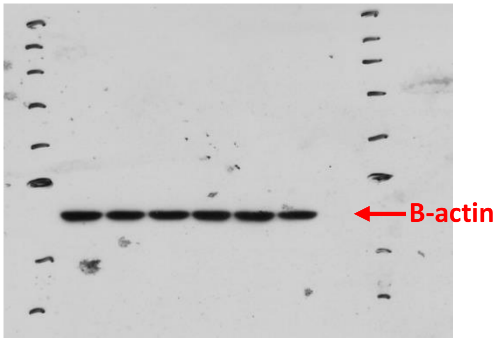

(PPAR-γ, n=3, 1:500, mouse monoclonal, Santacruz, #sc-7273)

(β-actin, n=3, 1:5,0000, mouse monoclonal, Santacruz, #sc-56459)

(l1=ND, l2=control, l3=WFA 0.25μM, l4= WFA 0.5μM, l5= WFA 1μM, l6=RSG 2 μM)

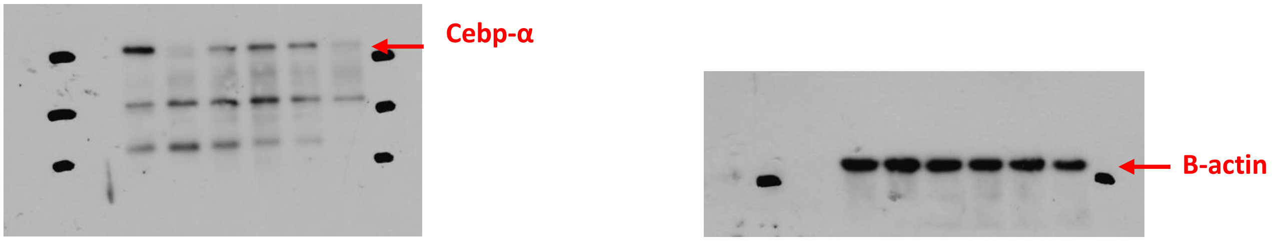

(Cebp-α, n=1, 1:1,000, rabbit polyclonal, Santacruz, #sc-61).

(β-actin, n=1, 1:5,0000, mouse monoclonal, Santacruz, #sc-56459)

(l1= RSG 2 µM, l2= WFA 1µM, l3=WFA 0.5 µM , l4= WFA 0.25µM, l5= control, l6=ND)

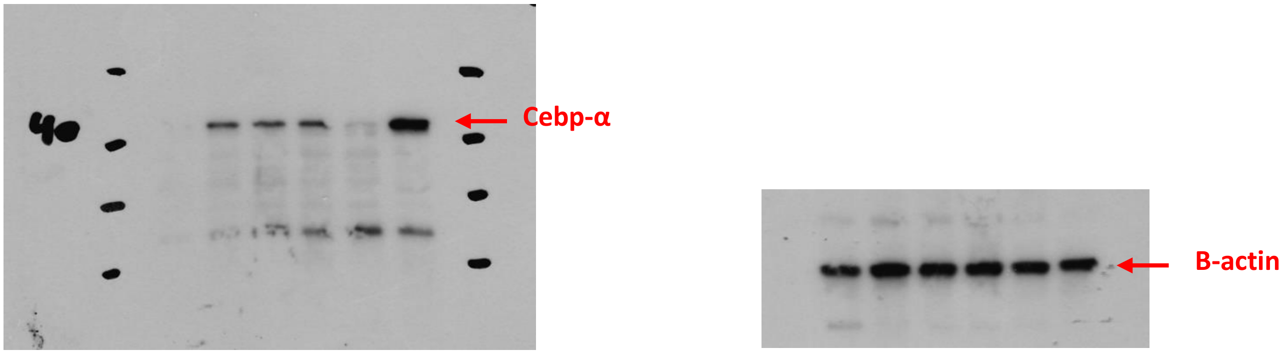

(Cebp-α, n=2, 1:1,000, rabbit polyclonal, Santacruz, #sc-61).

(β-actin, n=2, 1:5,0000, mouse monoclonal, Santacruz, #sc-56459)

(l1=ND, l2=control, l3=WFA 0.25µM, l4= WFA 0.5µM, l5= WFA 1µM, l6=RSG 2 µM)

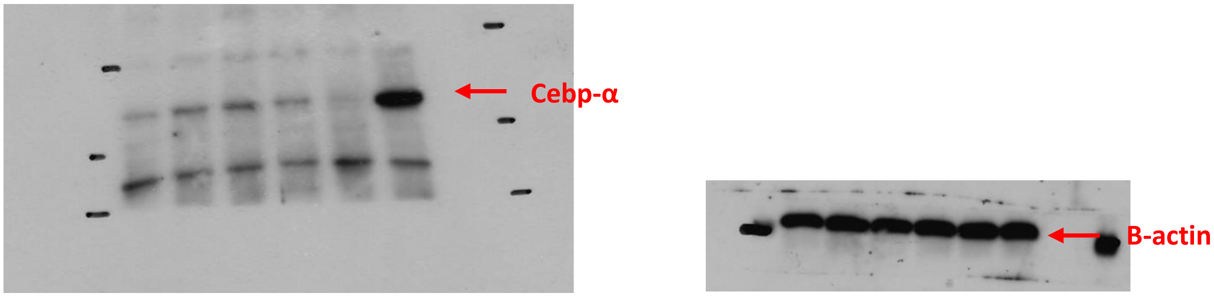

(Cebp-α, n=3, 1:1,000, rabbit polyclonal, Santacruz, #sc-61).

(β-actin, n=3, 1:5,0000, mouse monoclonal, Santacruz, #sc-56459)

(l1=ND, l2=control, l3=WFA 0.25µM, l4= WFA 0.5µM, l5= WFA 1µM, l6=RSG 2 µM)
